# Potentiated zinc and monovalent copper oxide as dietary supplements for weanling piglets: effects on systemic and mucosal immunity, gut permeability, and fecal microbiota composition

**DOI:** 10.3389/fvets.2025.1647844

**Published:** 2026-01-19

**Authors:** Luca Marchetti, Raffaella Rebucci, Paola Cremonesi, Filippo Biscarini, Bianca Castiglioni, Davide Lanzoni, Alessandra Monteiro, Yron Joseph Yabut Manaig, Valentino Bontempo

**Affiliations:** 1 Department of Veterinary Medicine and Animal Sciences (DIVAS), University of Milan, Lodi, Italy; 2 National Research Council, Institute of Agricultural Biology and Biotechnology (IBBA-CNR), Lodi, Italy; 3 Animine, Annecy, France

**Keywords:** trace elements, intestinal barrier, immune response, weaning, gut microbiome, animal health, gut health, nutrition

## Abstract

This study examines the potential of modulating gut health parameters in piglets by varying zinc/copper ratios administered through specialty oxide sources. A total of 84 piglets were selected after weaning and divided into four experimental treatment groups; the trial lasted 28 days. During the initial phase (1–14 d), the positive control (PC) received 2500 ppm of zinc, provided as conventional zinc oxide. In parallel, three additional treatment groups were formed, in which copper (Cu) and zinc (Zn) were supplemented using potentiated zinc oxide (Pot-ZnO) and monovalent copper oxide (Cu_2_O), at both European and Non-European recommended inclusion levels: EU (120 ppm of Zn; 140 ppm of Cu), Non-EU^+^ (300 ppm of Zn; 200 ppm of Cu), and Non-EU^−^ (300 ppm of Zn; 140 ppm of Cu). Lower Zn/Cu ratios characterized the second phase (15–28 d). Growth performance and fecal score were monitored throughout the trial. Blood samples were collected on days 1 and 14 from one subject per replicate to assess serum proinflammatory cytokines, immunoglobulins, and biomarkers of intestinal permeability. On day 28, one subject per replicate was slaughtered to obtain jejunal mucosa for the determination of jejunal secretory immunoglobulin A (sIgA) and alkaline phosphatase. Fecal samples collected on days 14 and 28 were used to analyze Zn and Cu content and to evaluate microbiota composition. A better fecal score was detected on day 4 in PC group compared to EU (*p* < 0.01). Serum immunoglobulin A increased in Non-EU^−^ vs. PC group (*p* < 0.05) at day 14. Serum diamine oxidase decreased in the PC and Non-EU^+^ groups vs. Non-EU^−^ group (*p* < 0.05) at day 14. sIgA increased in PC vs. Non-EU^−^ group (*p* < 0.01) at day 28. Zn was higher in PC fecal samples (*p* < 0.01), whereas fecal Cu increased in EU and Non-EU^+^ treatments at day 14 (*p* < 0.05). Analysis of fecal microbiota performed at day 14 showed decreased. Observed, Shannon, and Simpson metrics in the Non-EU^−^ group compared to the EU group (*p* < 0.05). Beta diversity highlighted a significant separation among groups at day 14 (*p* < 0.01). Differential abundance analysis revealed notable changes in genera composition among PC and EU groups at day 14 (*p* < 0.05). In conclusion, balanced Pot-ZnO and Cu_2_O administered at inclusion levels compliant with European levels of inclusion (EU) represent a valid strategy to enhance gut health of piglets during the first two weeks after weaning.

## 1 Introduction

The weaning period represents one of the most critical phases in pig production, during which the combined stress of social, nutritional, and physiological changes predisposes piglets to post-weaning diarrhea (PWD). This can compromise the development of intestinal microbiota, with long-term effects on gut health and growth of piglets (1). Thus, to avoid these complications, the administration of Zn and Cu in pharmacological dosages (2500/3000 mg/kg of Zn and up to 250 mg/kg of Cu) through zinc oxide (ZnO) and copper sulphate (CuSO_4_) has been extensively studied in the past (2, 3).

Generally, trace elements are supplied in piglet diets through commodities characterized by low bioavailability, which can have potential detrimental effects on the environment (4). Due to this, the European community promoted the ban on pharmacological dosages of Zn and remodulated the maximum permitted level of inclusion of Cu in postweaning piglets’ feeds. Thus, in Europe, Zn is permitted at a maximum inclusion level of 150 mg/kg in piglet diets during the weaning period, whereas Cu can be added at levels of up to 150 mg/kg in complete feed during the first four weeks after weaning, and up to 100 mg/kg from week five to week eight post-weaning (5). However, China still allows the administration of 1600 mg/kg of Zn during the first 2 weeks after weaning, while before 2018, up to 250 mg/kg Cu was allowed to ensure growth-promoting effects (6, 7). Following ingestion, ZnO and CuSO_4_ dissociate into their respective ions, which participate in distinct metabolic pathways and key enzymatic reactions. In particular, Zn ions may exert a direct catalytic function or act as structural stabilizers in several enzymatic processes, especially those involving metalloenzymes (8). Moreover, copper ions are well recognized for their role as free radical scavengers and regulators of iron metabolism (9). Furthermore, the high affinity of these ions for metalloproteins, particularly metallothioneins, is considered one of the key factors regulating homeostasis of trace elements (10). In addition, it was recently reported that particularly high Zn dosages can enhance metallothionein expression, directly impacting Cu metabolism in weaned piglets (11, 12). Thus, high dosages of zinc can negatively influence the absorption and bioavailability of other trace elements and nutrients (13).

In recent years, sources of trace elements with higher bioavailability have been extensively investigated. The enhanced bioavailability of specialty oxide sources can be attributed to their higher porosity, which increases the proportion of surface-exposed atoms and allows for a reduced dietary inclusion rate of the trace element (14). For instance, potentiated zinc oxide (Pot-ZnO) represents a processed form of ZnO with a free surface area 10- to 15-fold greater than conventional ZnO, due to its porosity. This increased surface exposure results in a porous formulation composed of smaller aggregated and agglomerated particles, characterized by enhanced bioavailability (15, 16). Conversely, monovalent copper oxide formulation (Cu_2_O) represents an alternative to CuSO_4_ and has been shown to improve growth performance in both piglets and broilers when administered at extranutritional levels. It also results in lower hepatic copper accumulation (17). Supplementing Zn and Cu through specialty oxide sources demonstrated positive effects in terms of bioavailability, gut health, and immunity. Pot-ZnO improved the expression of anti-inflammatory pathways and showed positive effects on growth performance, immunity, and gut health when administered to postweaning piglets (18–20). In contrast, the administration of Cu_2_O or alternative formulations such as tribasic copper chloride improved the performance of weaned piglets while exerting a bactericidal effect on gram-negative pathogens (7, 17, 21). Furthermore, several alternative trace element formulations have been proposed as potential modulators of the intestinal microbiota in piglets, thereby supporting gut health during the weaning period (21–23).

To the best of our knowledge, no studies have specifically addressed the impact of different Zn/Cu ratios supplemented through specialty oxide sources on the gut health of weanling piglets. Moreover, considering the dose-dependent effects of Zn and Cu, the available literature indicates that the reported outcomes were achieved at inclusion levels exceeding 150 mg/kg of complete feed, which are above the limits established in Europe. Therefore, the objective of this study was to evaluate the effects of different ratios of Pot-ZnO and Cu_2_O on systemic and local immunity, gut permeability markers, and fecal microbiota of weanling piglets.

## 2 Materials and methods

### 2.1 Experimental design and animal housing

Immediately after weaning, at 28 days of age (corresponding to day 1 of the trial), a total of 84 crossbreed Topigs piglets, half males and half females (7.14 ± 0.92 kg), were selected from “Azienda Agricola Arioli-Sangalli” (Genzone, Pavia, Italy) and transported to the experimental facilities of the Department of Veterinary Medicine and Animal Sciences of the University of Milan.

The trial was conducted using a randomized block design based on body weight. Piglets were allocated to four experimental treatments. Each treatment consisted of an equal number of homogeneous replicates. Piglets were divided into seven replicates per treatment with three subjects for each replicate (pen). They were kept in a single room with controlled environmental conditions. Piglets were organized in four groups (Table 1): positive control (PC), European levels of inclusion of Zn and Cu (EU), non-European levels of inclusion of Zn and Cu (Non-EU^+^), and non-European levels of inclusion of Zn group (Non-EU^−^).

**Table 1:** Experimental design of the trial.

Dietary treatments	Phase 1 (1–14 d)	Phase 2 (15–28 d)
PC	2500 mg/kg of Zn (ZnO) 0 mg/kg of Cu	150 mg/kg of Zn (ZnO) 150 mg/kg of Cu (CuSO_4_)
EU	120 mg/kg of Zn (Pot-ZnO) 140 mg/kg of Cu (Cu_2_O)	120 mg/kg of Zn (Pot-ZnO) 140 mg/kg of Cu (Cu_2_O)
Non-EU^+^	300 mg/kg of Zn (Pot-ZnO) 200 mg/kg of Cu (Cu_2_O)	150 mg/kg of Zn (Pot-ZnO) 200 mg/kg of Cu (Cu_2_O)
Non-EU^−^	300 mg/kg of Zn (Pot-ZnO) 140 mg/kg of Cu (Cu_2_O)	150 mg/kg of Zn (Pot-ZnO) 140 mg/kg of Cu (Cu_2_O)

PC group was fed the basal diet supplied with ZnO (72% of Zn) and CuSO_4_ (25% of Cu) formulations, while the remaining groups were fed potentiated zinc oxide (Pot-ZnO, HiZox^®^ 75% of Zn) and monovalent copper oxide (Cu_2_O, CoRouge^®^, 75% of Cu). As illustrated in Table 1, the experiment was divided into two phases: phase 1 (1–14 d) included higher dosages of Zn and Cu, while phase 2 (15–28 d) was characterized by the administration of lower Zn and Cu dosages. The cited compounds were provided by Animine (10 Rue Léon Rey Grange, 74,960 Annecy, France).

The temperature was set at 28 °C on day 1 and regulated weekly until reaching 24 °C at the end of the trial. Relative humidity was maintained below 65%, and the airflow was set at 10 m^3^/animal/h. Water and feed were available ad libitum from day 1 of the trial. Animals were allocated to pens with plastic grating flooring and 1.20 m^2^ of free surface. The trial lasted 28 days.

As reported in Table 2, weanling piglets were fed a unique basal diet formulated to satisfy the nutrient requirements suggested by the National Research Council (24). Feed samples (*n* = 5 for each phase) were analysed for dry matter (DM), crude protein (CP), crude fiber (CF), ether extract (EE), and ash, according to Association of Official Analytical Chemists (AOAC) official methods (25). Cu and Zn concentrations in feed were determined by an inductively coupled plasma (ICP) emission spectrometer (OPTIMA 3300 XL, Perkin-Elmer Corp., Waltham, MA, United States) as described in Xue et al. (26). Results of feed analysis are displayed in Tables 3, 4.

**Table 2:** Composition of weanling piglets’ diet.

Ingredients, % as fed	
Wheat meal	18.48
Extruded wheat	17.00
Barley meal	15.80
Bakery by-products	9.00
Dehulled flacked barley	8.80
Extruded soybean	6.90
Sweet whey	5.28
Soybean meal 48%	4.00
Flacked maize	4.00
Herring meal	2.60
soy protein concentrate (CP 52%)	1.50
Soybean hulls	1.40
Animal fat	1.00
L-Lysine	0.74
Soybean oil	0.60
Calcium formate	0.50
Dicalcium phosphate	0.37
L-Threonine	0.335
DL-Methionine	0.214
Sodium chloride	0.200
Calcium sulphate	0.190
L-tryptophan	0.091
Vitamin and trace elements premix^a^	1.00
Calculated nutrients values (% as fed)^b^	
DM, %	88.89
CP, %	16.70
EE, %	5.34
CF, %	3.00
Zn, mg/kg	15.00
Cu, mg/kg	6.00
NE, kcal/kg	2473
Lysine, %	1.34

a Supplements (per kg as fed): Vitamin A: 10,000 IU; Vitamin D3: 1,000 IU; Vitamin E: 50 mg; Vitamin B1:1.0 mg; VitamineB2: 3.0 mg; Vitamin B12: 0.02 mg; Vitamin B6: 3,0 mg; Pantothenic acid: 10 mg; Nicotinic acid: 15 mg; Biotin: 0.06 mg; Vitamin PP: 0,35 mg; Folic acid: 0,99 mg; Vitamin K3: 2 mg; Choline: 300 mg; Fe: 100 mg; Co: 0.75 mg; Mn: 10 mg; I: 0.75 mg; Se: 0.4 mg.

b DM: dry matter; CP: crude protein; EE: ether extract; CF: crude fiber; Zn: zinc; Cu: copper NE: net energy.

**Table 3:** Nutrient composition of experimental diets during phase 1 (1–14 d, *n* = 5 per treatment).

Phase 1 (1–14 d)	Dietary treatments^b^			
Parameters^a^, % as fed	PC	EU	Non-EU^+^	Non-EU^−^
DM	88.87 ± 3.83	88.81 ± 3.85	88.89 ± 3.82	88.88 ± 3.89
CP	16.81 ± 0.79	16.55 ± 0.78	16.23 ± 0.76	16.43 ± 0.75
EE	5.71 ± 0.35	5.85 ± 0.36	5.61 ± 0.35	5.74 ± 0.35
CF	3.15 ± 0.21	3.27 ± 0.24	3.22 ± 0.24	3.28 ± 0.26
Ash	4.33 ± 0.28	3.97 ± 0.26	4.01 ± 0.26	3.94 ± 0.26
Zn, mg/kg	2405.26 ± 220.05	131.34 ± 26.02	303.37 ± 44.09	309.12 ± 42.17
Cu, mg/kg	6.51 ± 1.60	135.56 ± 21.32	212.27 ± 33.08	129.37 ± 19.12

a DM: dry matter; CP: Crude protein; EE: ether extract; CF: crude fiber; Cu: copper; Zn: zinc. Data are presented as mean ± standard deviation.

b During phase 1 (1–14 d) the following Zn and Cu dosages were considered: PC = 2500 mg/kg of Zn (conventional ZnO); EU = 120 mg/kg of Zn (Pot-ZnO) and 140 mg/kg of Cu (Cu_2_O); Non-EU^+^ = 300 mg/kg of Zn (Pot-ZnO) and 200 mg/kg of Cu (Cu_2_O); Non-EU^−^ = 300 mg/kg of Zn (Pot-ZnO) and 140 mg/kg of Cu (Cu_2_O).

**Table 4:** Nutrient composition of experimental diets during phase 2 (15–28 d, *n* = 5 per treatment).

Phase 2 (15–28 d)	Dietary treatments^b^			
Parameters^a^, % as fed	PC	EU	Non-EU^+^	Non-EU^−^
DM	88.70 ± 3.60	88.83 ± 3.82	88.94 ± 3.74	88.97 ± 3.86
CP	16.58 ± 0.74	16.32 ± 0.77	16.43 ± 0.71	16.62 ± 0.78
EE	5.68 ± 0.33	5.81 ± 0.38	5.45 ± 0.30	5.51 ± 0.37
CF	3.10 ± 0.21	3.21 ± 0.24	3.15 ± 0.23	3.27 ± 0.25
Ash	4.31 ± 0.26	4.02 ± 0.24	3.98 ± 0.24	3.96 ± 0.27
Zn, mg/kg	132.11 ± 26.17	114.15 ± 21.43	141.18 ± 28.56	152.16 ± 23.02
Cu, mg/kg	139.12 ± 22.02	128.16 ± 24.05	182.13 ± 34.03	143.11 ± 25.04

a DM: dry matter; CP: Crude protein; EE: ether extract; CF: crude fiber; Cu: copper; Zn: zinc. Data are presented as mean ± standard deviation (SD).

b During phase 2 (15–28 d) the following Zn and Cu dosages were considered: PC = 150 mg/kg of Zn (conventional ZnO) and 150 mg/kg of Cu (conventional CuSO_4_); EU = 120 mg/kg of Zn (Pot-ZnO) and 140 mg/kg of Cu (Cu_2_O); Non-EU^+^ = 150 mg/kg of Zn (Pot-ZnO) and 200 mg/kg of Cu (Cu_2_O); Non-EU^−^ = 150 mg/kg of Zn (Pot-ZnO) and 140 mg/kg of Cu (Cu_2_O).

### 2.2 Fecal score and growth performance evaluation

The fecal score was evaluated from day 1 to 28, using a Bristol stool scale from 1 (normal) to 7 (severe diarrhea) (27). Piglets were weighed on days 1, 14, and 28 to assess body weight (BW) and average daily gain (ADG). Contextually, removable feed trays were weighed to further calculate the average daily feed intake (ADFI), the feed conversion ratio (FCR), and the feed efficiency (FE).

### 2.3 Proinflammatory cytokines, immunoglobulins, and intestinal permeability markers assessment

One subject per replicate was selected based on the average body weight of the pen at the start of the trial for blood sampling. Therefore, on days 1 and 14, blood samples were obtained from the selected subjects by jugular venipuncture using a 20G needle (VACUETTE^®^, Greiner Bio-One GmbH) and a Vacutainer red-top tube coated with microscopic silica particles (10 mL). Serum aliquots were obtained through centrifugation at 3000 rpm for 15 min and then stored at −20 °C until analysis. Interleukins 6 and 1β (IL-6 and IL-1β) and immunoglobulins A, G, and M (IgA, IgG, and IgM) were analyzed using enzyme-linked immunosorbent assays (Immunological Sciences, Società Italiana Chimici, Rome, IT). Diamine oxidase (DAO) and L-lactate were analyzed using colorimetric assays (Immunological Sciences, Società Italiana Chimici, Rome, IT) to assess gut permeability.

### 2.4 Jejunal secretory immunoglobulins A (sIgA) and alkaline phosphatase (ALP) quantification

At the end of the trial, animals previously selected for blood sampling were slaughtered to collect the jejunal mucosa. The small intestine was removed, and the jejunum was promptly isolated and flushed with ice-cold phosphate-buffered saline (PBS). The mucosa was gently scraped using a slide to obtain mucosal samples, which were frozen in liquid nitrogen and stored at −80 °C. Jejunal mucosal aliquots (100 mg) were prepared in 2 mL microtubes (Sarstedt AG & Co) and homogenized in 1 mL of PBS. Secretory IgA and ALP levels were then quantified using ELISA kits (Immunological Sciences, Società Italiana Chimici, Rome, IT).

### 2.5 Zinc and copper quantification in fecal samples

After collecting fecal samples on days 14 and 28 from the selected subjects, the concentration of fecal trace elements was determined using the methodology depicted by Zhuo et al. (28). In total, 50 mg of sample was added to a Teflon tube prepared with 8 mL of concentrated nitric acid. A 20 min microwave digestion at 180 °C was performed. After cooling the system, samples were diluted with deionized water. Finally, Zn and Cu concentrations were determined by inductively coupled plasma optical emission spectrometry (ICP-OES) (OPTIMA 3300 XL, Perkin-Elmer Corp., Waltham, MA, United States).

### 2.6 16S rRNA gene sequencing

Fecal samples were collected at days 14 and 28 in sterile vials from 28 piglets (7 PC, 7 EU, 7 Non-EU^+^, and 7 Non-EU^−^) and stored at −80 °C until DNA extraction. The DNA was extracted from each sample using the QIAmp Fecal Pro kit (Qiagen, Hilden, Germany), according to the manufacturer’s protocol. DNA quality and quantity were assessed using a NanoDrop ND-1000 spectrophotometer (NanoDrop Technologies, Wilmington, DE, United States). The extracted DNA was stored at −20 °C. Bacterial DNA was amplified using the primers described by Caporaso et al. (29), which target the V3-V4 hypervariable regions of the 16S rRNA gene. All PCR amplifications were performed in 25 μL volumes per sample. A total of 12.5 μL of KAPA HIFI Master Mix 2 × (Kapa 344 Biosystems, Inc., MA, United States) and 0.2 μL of each primer (100 μm) were added to 2 μL of genomic DNA (5 ng/μL). Blank controls (no DNA template added to the reaction) were also assessed. The first amplification step was performed in an Applied Biosystem 2,700 thermal cycler (ThermoFisher Scientific, MA, United States). Samples were denatured at 95 °C for 3 min, followed by 25 cycles consisting of denaturation at 98 °C for 30 s, annealing at 56 °C for 1 min, and extension at 72 °C for 1 min, with a final extension at 72 °C for 7 min. Amplicons were cleaned with Agencourt AMPure XP (Beckman, Coulter Brea, CA, United States), and libraries were prepared following the 16S Metagenomic Sequencing Library Preparation Protocol (Illumina, San Diego, CA, United States). The libraries obtained were quantified by real-time PCR with KAPA Library Quantification Kits (Kapa Biosystems, Inc., MA, United States), pooled in equimolar proportion, and sequenced in a single MiSeq (Illumina, San Diego, CA, United States) run with 2 × 250-base paired-end reads.

### 2.7 Bioinformatics processing

Demultiplexed paired-end reads from 16S rRNA-gene sequencing were first checked for quality using FastQC (30). Reads were then cleaned by removing primers and adapters using the Python tool Cutadapt and by trimming for quality with the C++ tool Sickle at a Phred threshold > 20 (i.e., the terminal part of the reads was removed if of low quality) (31, 32). After quality filtering, forward and reverse paired-end reads were merged using the MICCA (Microbial Community Analysis) Python pipeline, specifically the “mergepairs” function with default parameters (i.e., minimum overlap length = 32 bp; maximum number of mismatches in the overlap region = 8) (33). As reads were filtered for quality, and reads with missing/uncalled bases or with an expected error rate larger than 1% (1 error in 100 bases) were discarded. All remaining reads were used to identify Operational Taxonomic Units (OTUs) using a denoising approach implemented in the MICCA function “ut” (method used: “denovo_unoise”) (34). Finally, the identified OTUs were classified using the MICCA function “classify” to assign taxa as annotated in the SILVA132 reference database using the following parameters: maximum number of hits—taxa—to consider for each out = 3; assigning of taxon if present in at least 0.50 of the hits; rejecting OTU if the fraction of alignment to the reference sequence was lower than 0.75 (35). The obtained OTU table was filtered by removing the least represented OTUs with < 20 counts in fewer than four samples.

### 2.8 Alpha and beta diversity

Fecal microbial diversities were assessed within samples (alpha diversity) and between samples (beta diversity). All indexes for alpha were estimated from the complete OTU, filtered for OTUs with more than 10 total counts distributed in at least two samples, and normalized for uneven sequencing depth by cumulative sum scaling (CSS). Within-sample microbial richness and diversity were estimated using the following indices: Chao1 and Abundance-based Coverage Estimator (ACE) for richness, Shannon and Simpson for evenness, and Fisher’s alpha for diversity. The across-sample microbiota diversity was assessed using Bray–Curtis dissimilarity. Among groups and pairwise, Bray-Curtis dissimilarities were determined non-parametrically using the permutational analysis of variance approach (999 permutations).

### 2.9 Statistical analysis

Fecal score and growth performance were analyzed using a one-way ANOVA, which was performed using the GLM procedure of SAS software (version 9.4; SAS Institute Inc., Cary, NC, United States). Proinflammatory cytokines, immunoglobulins, gut permeability markers, fecal Zn and Cu content, and jejunal sIgA and ALP activity were also evaluated using the GLM procedure of SAS. Normal distribution of data was tested using the Shapiro–Wilk test. When data were not normally distributed, the Kruskal–Wallis test was utilized. Homogeneity of variances across groups was verified using Levene’s test, and when significant heterogeneity of variances was detected, the Welch test was performed to adjust the degrees of freedom. Post-hoc comparisons among groups were carried out using Tukey’s test (for parametric analyses) or Dunn’s test (for non-parametric analyses). Pens were considered the experimental units. The comparison of alpha diversity and OTU abundance between experimental groups was evaluated using the following linear model:where: y_ijk is the alpha diversity index value or OTU abundance for sample i from treatment k at timepoint j; timepoint is the effect of time (day 14 or 28); treatment is the effect of the dietary treatment (PC, EU, Non-EU^+^, and Non-EU^−^); e_ijk are the model residuals. Differences in community structure were assessed using permutational multivariate analysis of variance based on Bray–Curtis dissimilarities. To evaluate the significance of variance partitioning across timepoints and treatments, the analysis was conducted with 999 permutations. Statistical significance was set at *p*-value < 0.05 and high significance at p-value < 0.01 for all the considered evaluations.


$$\begin{array}{l} y\_\mathrm{ijk}=\mu +\beta \_1*\text{timepoint}\_j\\ +\beta \_2*\text{treatment}\_k+e\_\mathrm{ijk} \end{array}$$

## 3 Results

### 3.1 Fecal score

**Figure 1:**
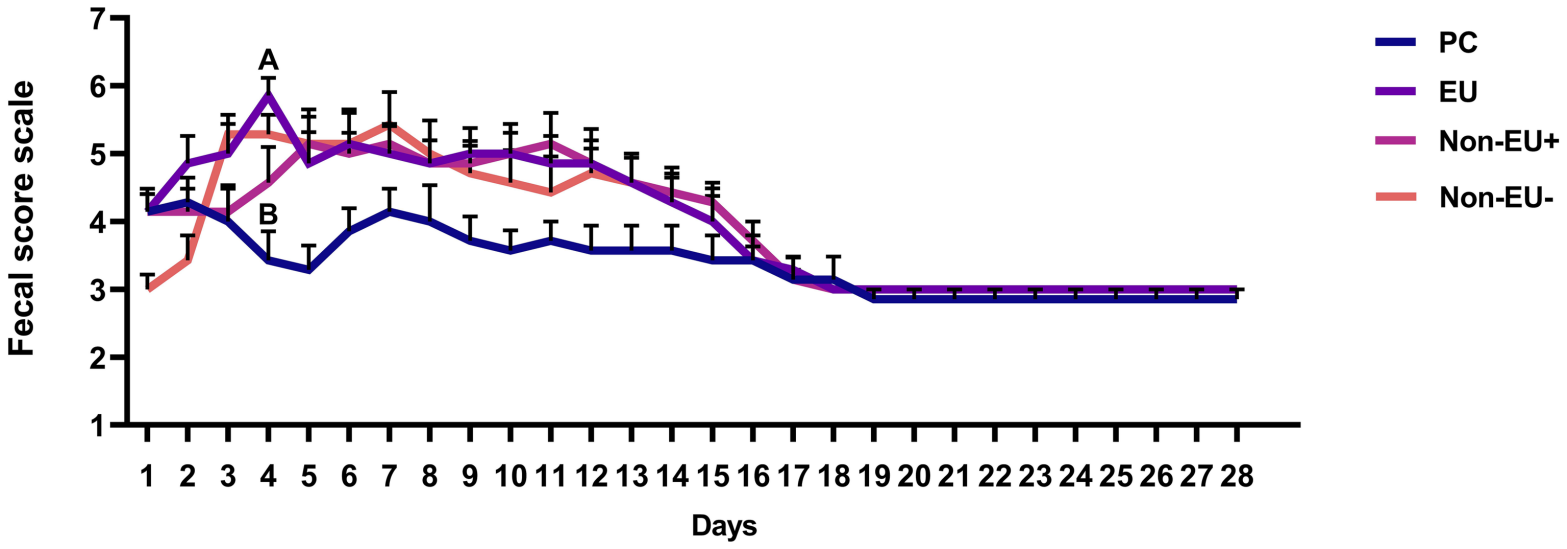
Fecal score registered during the trial through a Bristol stool scale (*n* = 7 per group). Different letters mark statistically significant results between groups (A, B; *p* < 0.01). Data are presented as mean ± standard error mean (SEM). EU revealed a higher fecal score than PC at day 4 of the trial. The lack of differences among groups throughout most of the trial period indicates that the tested ratios of Pot-ZnO and Cu_2_O could improve fecal score to a degree comparable with pharmacological ZnO administration.

As reported in Figure 1, the only significant difference between PC and EU was found on day 4 (*p* < 0.01). No differences among groups were detected throughout the trial, resulting only in numerically lower fecal score values in the PC group compared to EU, Non-EU^+^, and Non-EU^−^ groups from day 4 to day 15 of the trial.

### 3.2 Growth performance

As reported in Table 5, BW did not differ among groups on days 1, 15, and 28 of the trial. Likewise, ADG showed no significant differences among treatments during either phase 1 (days 1–14) or 2 (days 15–28), resulting in comparable overall growth performance across the entire experimental period (days 1–28). Similarly, ADFI did not vary among the four treatment groups when data were analyzed for each phase or for the overall study duration. Consequently, FCR and FE were also unaffected by dietary treatment in both phases and over the entire course of the trial.

**Table 5:** Performances of pens registered during the trial (*n* = 7 per group).

Parameters^a^	Dietary treatments^b^^,^^c^			
	PC	EU	Non-EU^+^	Non-EU^−^
BW, kg				
1 d	22.60 ± 2.47	22.55 ± 2.72	22.61 ± 1.70	22.60 ± 2.47
14 d	32.82 ± 4.38	31.55 ± 3.67	31.55 ± 1.87	31.10 ± 2.89
28 d	50.57 ± 6.14	50.43 ± 4.69	51.01 ± 2.67	51.59 ± 3.54
ADG, kg/d				
1–14 d	0.73 ± 0.15	0.64 ± 0.11	0.64 ± 0.12	0.61 ± 0.06
15–28 d	1.27 ± 0.15	1.35 ± 0.13	1.39 ± 0.14	1.46 ± 0.13
1–28 d	0.99 ± 0.14	0.99 ± 0.10	1.01 ± 0.07	1.03 ± 0.07
ADFI, kg/d				
1–14 d	1.27 ± 0.26	1.18 ± 0.21	1.12 ± 0.18	1.19 ± 0.15
15–28 d	2.14 ± 0.39	2.28 ± 0.39	2.23 ± 0.24	2.34 ± 0.31
1–28 d	1.71 ± 0.32	1.73 ± 0.29	1.67 ± 0.16	1.77 ± 0.19
FCR				
1–14 d	1.75 ± 0.20	1.85 ± 0.23	1.79 ± 0.34	1.97 ± 0.25
15–28 d	1.67 ± 0.20	1.68 ± 0.16	1.61 ± 0.15	1.60 ± 0.17
1–28 d	1.70 ± 0.17	1.63 ± 0.16	1.65 ± 0.13	1.70 ± 0.11
FE				
1–14 d	0.58 ± 0.07	0.55 ± 0.07	0.58 ± 0.10	0.51 ± 0.06
15–28 d	0.60 ± 0.07	0.60 ± 0.06	0.63 ± 0.06	0.63 ± 0.06
1–28 d	0.59 ± 0.06	0.58 ± 0.05	0.61 ± 0.05	0.59 ± 0.04

a BW: body weight; ADG: average daily gain; ADFI: average daily feed intake; FCR: feed conversion rate; FE: feed efficiency. Data are presented as mean ± standard deviation (SD). Absence of letters indicates lack of statistically significant differences among groups (*p* > 0.05).

b During phase 1 (1–14 d) the following Zn and Cu dosages were considered: PC = 2500 mg/kg of Zn (conventional ZnO); EU = 120 mg/kg of Zn (Pot-ZnO) and 140 mg/kg of Cu (Cu_2_O); Non-EU^+^ = 300 mg/kg of Zn (Pot-ZnO) and 200 mg/kg of Cu (Cu_2_O); Non-EU^−^ = 300 mg/kg of Zn (Pot-ZnO) and 140 mg/kg of Cu (Cu_2_O).

c During phase 2 (15–28 d) the following Zn and Cu dosages were considered: PC = 150 mg/kg of Zn (conventional ZnO) and 150 mg/kg of Cu (conventional CuSO_4_); EU = 120 mg/kg of Zn (Pot-ZnO) and 140 mg/kg of Cu (Cu_2_O); Non-EU^+^ = 150 mg/kg of Zn (Pot-ZnO) and 200 mg/kg of Cu (Cu_2_O); Non-EU^−^ = 150 mg/kg of Zn (Pot-ZnO) and 140 mg/kg of Cu (Cu_2_O).

### 3.3 Proinflammatory cytokines, immunoglobulins, and intestinal permeability markers

**Figure 2:**
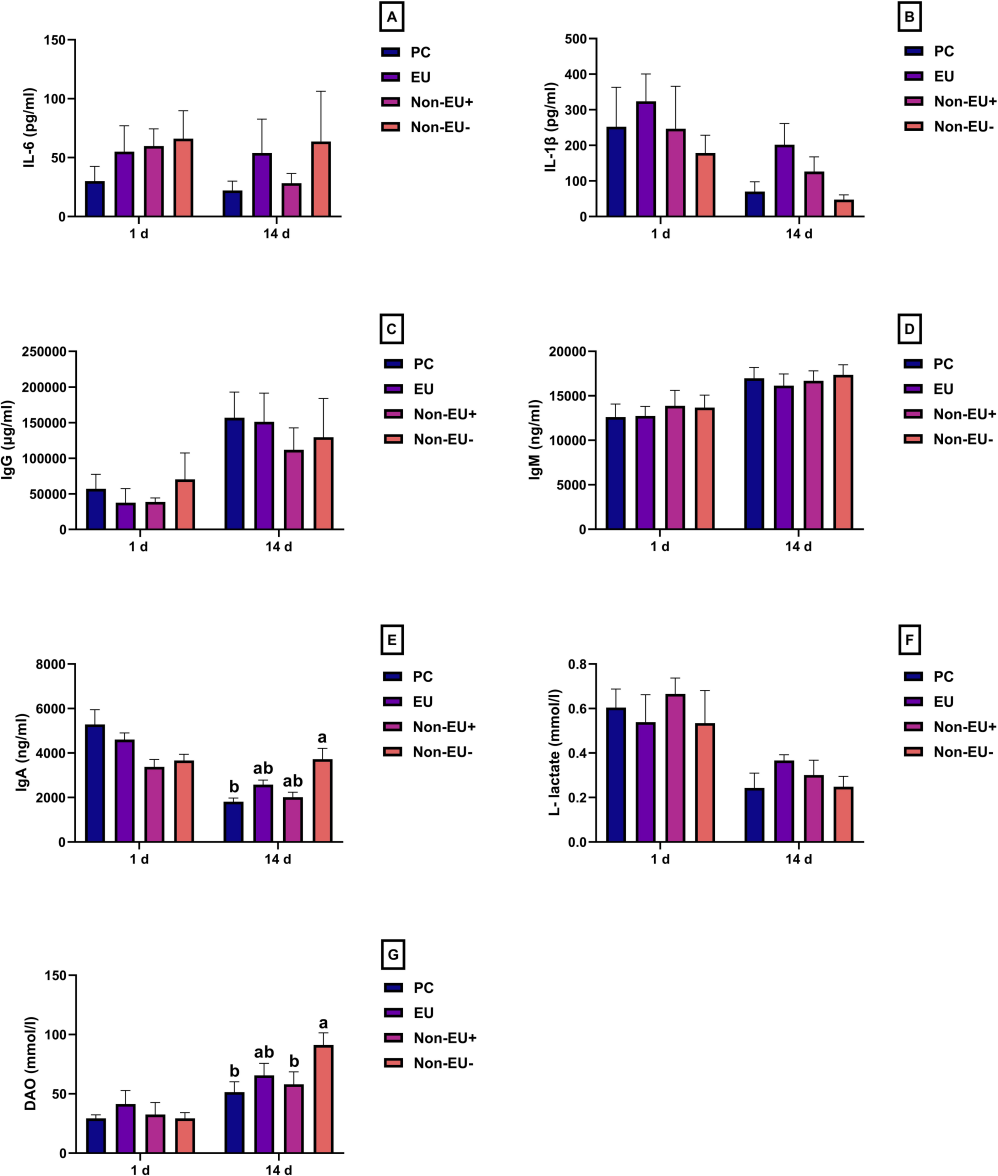
IL-6 **(A)**, IL-1β **(B)**, IgG **(C)**, IgM **(D)**, IgA **(E)**, L-lactate **(F)**, and DAO **(G)** were analyzed at days 1 and 14 during the trial (*n* = 7 per group). Data are presented as mean ± standard error mean (SEM). Different letters mark statistically significant results between groups (a, b; *p* < 0.05). No differences were found among groups in terms of circulating proinflammatory cytokine concentrations. While IgM and IgG were not different among groups, IgA was higher in Non-EU^−^ compared to PC. Therefore, the tested ratios of Pot-ZnO and Cu_2_O can efficiently modulate the systemic immune response of piglets when compared to a pharmacological administration of ZnO throughout weaning. DAO revealed to be higher in Non-EU^−^ rather than PC and Non-EU^+^. In addition, the EU group did not differ when compared to the PC and Non-EU^+^ groups. Results suggest that intestinal permeability may be positively influenced by a more balanced ratio of Zn and Cu when provided via Pot-ZnO and Cu_2_O.

Proinflammatory cytokines IL-6 and IL-1β were unaffected by the treatments during the trial (Figures 2A,B). Furthermore, the detected levels of serum IgG and IgM showed no significant differences among groups (Figures 2C,D). As indicated by Figure 2E, the Non-EU^−^ group recorded higher levels of serum IgA than the PC group (3723.78 ± 1086.52 ng/mL vs. 1808.11 ± 450.78 ng/mL; *p* < 0.05) on day 14 after weaning. However, no differences were found among PC, EU, and Non-EU^+^ when considering IgA levels.

No differences among groups were detected in serum L-lactate levels measured at days 1 and 14 after weaning (Figure 2F). On the contrary, DAO levels (Figure 2G) were significantly affected by an unbalanced administration of Cu and Zn through specialty oxide sources. The results showed an enhancement of DAO levels at day 14 in the Non-EU^−^ group compared to the PC group (91.16 ± 20.57 ng/mL vs. 51.43 ± 21.58 ng/m; *p* < 0.05, Figure 2G) and Non-EU^+^ group (91.16 ± 20.57 ng/mL vs. 58.00 ± 31.69 ng/mL; *p* < 0.05, Figure 2G). In contrast, no difference was detected in DAO levels among the PC, EU, and Non-EU^+^ groups.

### 3.4 Jejunal secretory immunoglobulin A and alkaline phosphatase

**Figure 3:**
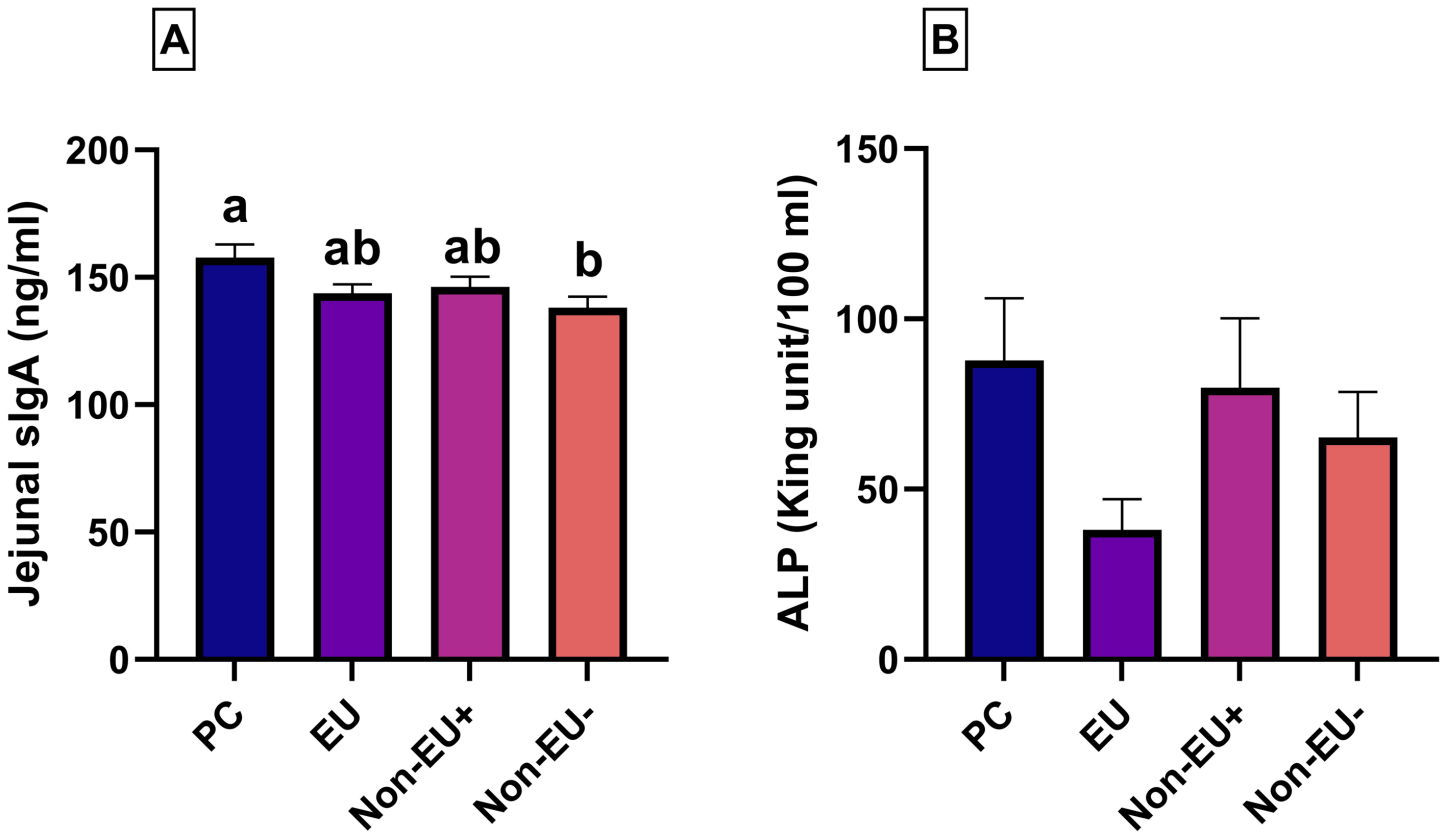
The levels of sIgA **(A)** and ALP **(B)** in the Jejunum were registered on day 28 of the trial (*n* = 7 per group). Different letters mark statistically significant results between groups (a, b; *p* < 0.01). Data are presented as mean ± standard error mean (SEM). Jejunal sIgA revealed to be higher in PC when compared to Non-EU^−^. No differences were found among EU, Non-EU^+^, and PC groups. These results suggest that a balanced supplementation of Pot-ZnO and Cu_2_O can positively influence local immunity markers.

Briefly, the PC group showed a higher level of sIgA than the Non-EU^−^ group (157.73 ± 19.58 ng/mL vs. 138.01 ± 16.88 ng/mL; *p* < 0.05, Figure 3A), and sIgA levels were similar among PC, EU, and Non-EU^+^ groups. Moreover, the jejunal ALP activity was similar among treatment groups at day 28 (Figure 3B).

### 3.5 Zinc and copper in fecal samples

**Figure 4:**
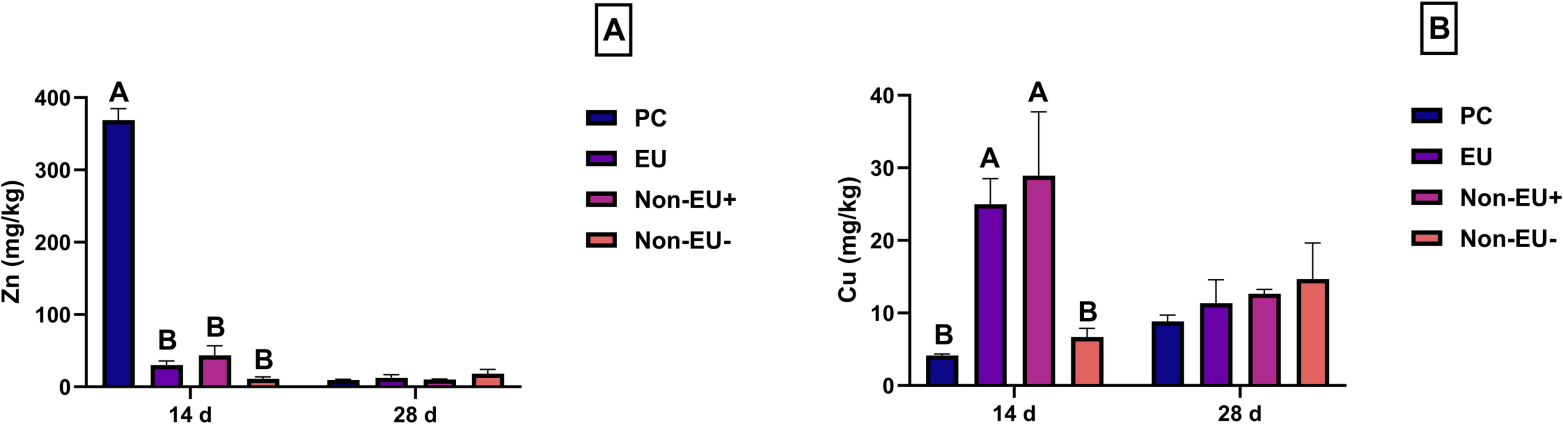
Zn and Cu levels were determined in fecal samples collected at days 14 and 28 of the trial (**A,B**, respectively). Different letters mark statistically significant results between groups (A, B; *p* < 0.01). Data are presented as mean ± standard error mean (SEM). As expected, Zn values detected at day 14 highlighted that the pharmacological supplementation of ZnO is less bioavailable, possibly leading to adverse effects in terms of environmental excretion. Cu values revealed to be higher in EU and Non-EU^+^ groups compared to the PC group. These results can be explained by the lack of Cu administration in PC animals. The differences existing between EU and Non-EU^−^ groups should be linked to the endogenous regulation of trace elements, which could influence Cu excretion.

Zinc levels in fecal samples collected at day 14 revealed that the PC group showed higher Zn concentration when compared to EU, Non-EU^+^, and Non-EU^−^ groups (368.76 ± 42.11 mg/kg vs. 30.04 ± 15.16 mg/kg, 43.53 ± 35.49 mg/kg, and 11.31 ± 6.24 mg/kg; *p* < 0.01, Figure 4A). The EU group showed higher levels of Cu at day 14 compared to the PC (25.00 ± 9.32 mg/kg vs. 4.16 ± 0.46 mg/kg; *p* < 0.01, Figure 4B) and Non-EU^−^ groups (25.00 ± 9.32 mg/kg vs. 6.69 ± 3.15 mg/kg; *p* < 0.01, Figure 4B). Moreover, the Non-EU^+^ group demonstrated higher copper concentrations than both PC and Non-EU^−^ groups at day 14 (28.92 ± 23.28 mg kg vs. 4.16 ± 0.46 mg/kg and 6.69 ± 3.15 mg/kg; *p* < 0.01, Figure 4B). No difference was detected among groups in terms of Zn and Cu fecal concentrations at day 28.

### 3.6 Fecal alpha and beta diversity

**Figure 5:**
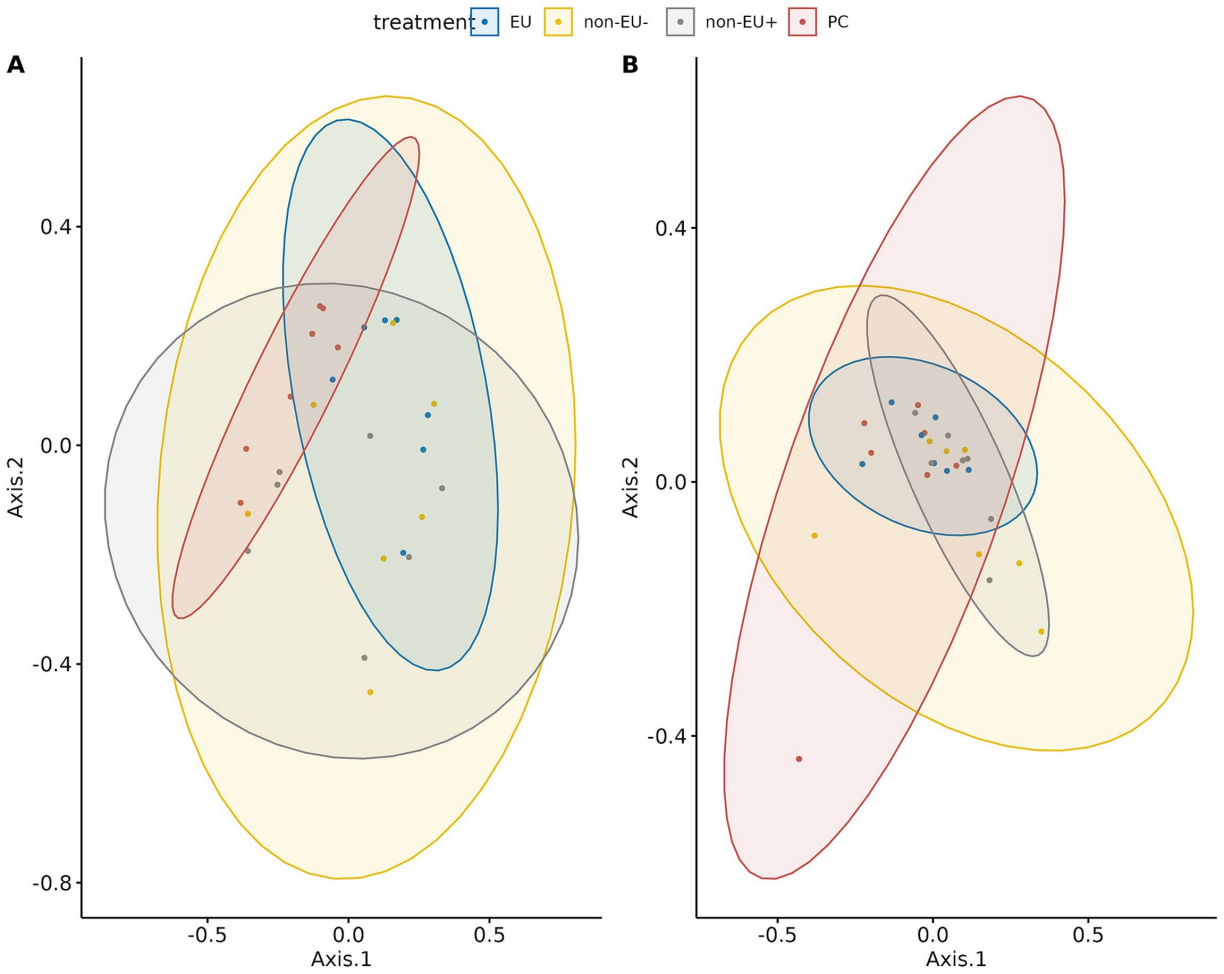
Beta diversity data referred to fecal samples analyzed at day 14 **(A)** and 28 **(B)**. As evidenced by the graphs, PC and EU treatment displayed a notable separation in terms of between-sample diversity.

EU fecal samples collected at day 14 showed higher values for observed, Shannon, and Simpson metrics compared to Non-EU^−^ group (*p* < 0.05, Table 6). Conversely, the EU group displayed a higher Simpson metric value than the Non-EU^+^ group (*p* < 0.05, Table 6). Fecal samples collected at day 28 revealed no effect of treatments on conditioning alpha diversity metrics. Data concerning beta diversity are presented in Figure 5. Fecal samples collected on day 14 showed a significant separation among treatments (*p* < 0.01, Figure 5A). Furthermore, no effects of treatments were evidenced from Permutational Multivariate Analysis of Variance (PERMANOVA) performed on fecal samples collected at day 28 (Figure 5B).

**Table 6:** Average counts of alpha diversity metrics in PC, EU, Non-EU^+^, and Non-EU^−^ samples (*n* = 7 per group).

Metrics^1,2,3^	PC	EU	Non-EU^+^	Non-EU^−^
14 d				
ACE	1808.8 ± 330.8	1974.8 ± 171.9	1744.7 ± 167.4	1620.1 ± 296.4
Chao1	1809.0 ± 337.9	1973.1 ± 172.4	1730.0 ± 170.5	1617.6 ± 299.3
Fisher	324.5 ± 48.2	347.3 ± 32.8	321.9 ± 32.3	301.0 ± 68.2
InvSimpson	67.7 ± 14.9	82.6 ± 20.9	52.6 ± 37.9	49.1 ± 26.9
Observed	1680.6 ± 315.6^ab^	1829.4 ± 165.1^a^	1607.3 ± 168.3^ab^	1467.4 ± 279.1^b^
Shannon	5.6 ± 0.1^ab^	5.7 ± 0.1^a^	5.4 ± 0.3^ab^	5.3 ± 0.3^b^
Simpson	0.985 ± 0.003^ab^	0.987 ± 0.003^a^	0.976 ± 0.010^b^	0.975 ± 0.010^b^
28 d				
ACE	1826.3 ± 318.5	2025.9 ± 249.0	2101.4 ± 309.7	1933.3 ± 250.6
Chao1	1822.1 ± 319.0	2021.0 ± 252.3	2107.0 ± 313.9	1927.8 ± 248.7
Fisher	333.4 ± 67.7	369.9 ± 45.0	374.2 ± 49.0	347.0 ± 51.7
InvSimpson	62.0 ± 15.2	63.1 ± 8.0	62.4 ± 21.6	58.8 ± 9.9
Observed	1672.0 ± 305.0	1870.9 ± 228.0	1930.9 ± 310.4	1786.7 ± 246.5
Shannon	5.5 ± 0.3	5.6 ± 0.2	5.6 ± 0.2	5.5 ± 0.2
Simpson	0.9 ± 0.004	0.9 ± 0.002	0.9 ± 0.004	0.9 ± 0.003

^1^Results referred to contrasts comparisons among alpha diversity metrics values of the four dietary treatments (PC, EU, Non-EU^+^, and Non-EU^−^) within time points (14 d and 28 d). Results are expressed as mean average count ± standard deviation (SD). Different letters within the same row indicate statistical significance (a,b; *p* < 0.05). ^2^During phase 1 (1–14 d) the following Zn and Cu dosages were considered: PC = 2500 mg/kg of Zn (conventional ZnO); EU = 120 mg/kg of Zn (Pot-ZnO) and 140 mg/kg of Cu (Cu_2_O); Non-EU^+^ = 300 mg/kg of Zn (Pot-ZnO) and 200 mg/kg of Cu (Cu_2_O); Non-EU^−^ = 300 mg/kg of Zn (Pot-ZnO) and 140 mg/kg of Cu (Cu_2_O). ^3^During phase 2 (15–28 d) the following Zn and Cu dosages were considered: PC = 150 mg/kg of Zn (conventional ZnO) and 150 mg/kg of Cu (conventional CuSO_4_); EU = 120 mg/kg of Zn (Pot-ZnO) and 140 mg/kg of Cu (Cu_2_O); Non-EU^+^ = 150 mg/kg of Zn (Pot-ZnO) and 200 mg/kg of Cu (Cu_2_O); Non-EU^−^ = 150 mg/kg of Zn (Pot-ZnO) and 140 mg/kg of Cu (Cu_2_O).

### 3.7 Differential abundances in fecal samples

**Figure 6:**
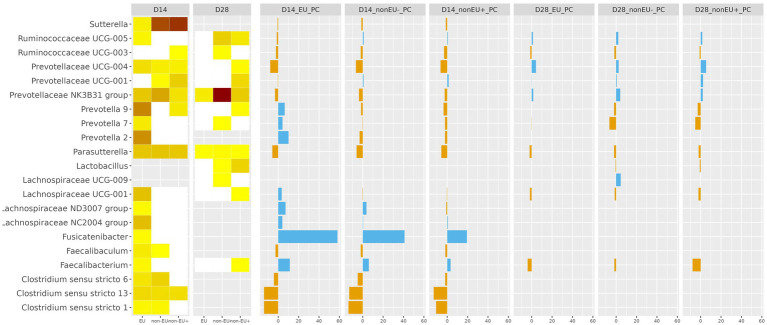
Differential abundances were determined in fecal samples collected at days 14 and 28. A significance level between *p* < 0.05 (light yellow) and *p* < 0.01 (dark brown) was considered for dietary treatments comparisons (D14 and D28 columns). The most appreciable differences were identified in EU vs. PC comparisons at day 14. The EU group evidenced enhanced abundances of *Prevotella*, *Lachnospiraceae*, and *Fusicatenibacter*, whereas *Sutterella* and *Clostridium sensu stricto* displayed reduced abundances.

Differential abundances of fecal samples collected at days 14 and 28 have been presented in Figure 6. At day 14, the EU samples showed a slight decrease in abundance of *Ruminococcaceae* and *Prevotellaceae* genera compared to the PC samples (*p* < 0.05). On the contrary, *Prevotella* genus abundance was higher in the EU group samples compared to the PC group (*p* < 0.05). Similarly, *Lachnospiraceae* abundance increased in the EU samples compared to the pharmacological administration of ZnO (*p* < 0.05). This trend was also maintained in *Fusicatenibacter* abundance, which was found to be higher in the EU samples than the PC samples (*p* < 0.05). *Sutterella* and *Parasutterella* were less represented in the EU, Non-EU^+^, and Non-EU^−^ treatment groups compared to the PC group (*p* < 0.05). In addition, *Clostridium sensu stricto* genera were less abundant in the EU, Non-EU+, and Non-EU^−^ samples than the PC samples (*p* < 0.05). However, analyses performed on samples collected at day 28 revealed that genus abundances were similar among the four treatment groups, which aligns with previous results regarding beta diversity.

## 4 Discussion

The aim of this study was to investigate the possibility of optimizing zinc and copper dietary administration through different ratios of Pot-ZnO and Cu_2_O to ensure optimal immunity, gut health, and intestinal microbiota development in weanling piglets. In the study, the registered fecal score revealed significant differences between PC and EU groups only at day 4 of the trial. The fecal score stayed unaffected by the treatments during the remaining days, and the lack of significant differences among treatment groups was in accordance with the available literature. Indeed, Peng et al. (20) did not find differences in diarrhea rate of early weaned piglets compared to a positive control group that was fed 3000 mg/kg of ZnO and two other groups fed 750 mg/kg and 1500 mg/kg of Pot-ZnO. Moreover, the dietary supplementation with 200 or 300 mg/kg of Pot-ZnO in weanling piglets resulted in similar or improved fecal consistency compared to the administration of 3000 mg/kg of conventional ZnO (36). In this study, the PC group displayed numerically lower fecal score values than the remaining groups throughout the trial. However, the results indicate that Pot-ZnO and Cu_2_O could improve fecal scores to a degree comparable to pharmacological ZnO administration.

Bonetti et al. (37) demonstrated that alternative forms of ZnO administered at non-European concentrations could increase or maintain piglet performance compared to pharmacological ZnO dosages. In contrast, Morales et al. (38) illustrated the capacity of Pot-ZnO to ameliorate the performance of weanling piglets in medium-low sanitary conditions when administered at 150 mg/kg. Moreover, Lei et al. (39) evidenced that the growth performance of weaned piglets was not different when comparing different inclusion levels of coated ZnO (from 300 mg/kg up to 2000 mg/kg) to a pharmacological administration of conventional ZnO (3000 mg/kg). In addition, Cu could positively influence the growth performance of piglets by stimulating lipase activity and lipid digestibility, enhancing ghrelin secretion in the stomach, and modulating intestinal microbiota (40). Low (50 mg/kg) and high (150 mg/kg) dosages of nano-Cu oxide supplementation in weanling piglets raised under heat stress conditions allow achievement of growth performance similar to piglets fed equal dosages of conventional CuSO_4_ (41). Our study aligns with previous observations, as growth performances did not differ among groups, demonstrating that the tested Pot-ZnO and Cu_2_O ratios maintain piglet growth comparable to a pharmacological dosage of ZnO.

IgA represents an important defense tool for the host to counterattack early infections (42). Moreover, serum IgA could be considered an indirect marker of intestinal health. Serum IgA levels could be related to intestinal mast cell activity, and intestinal junctions are disrupted during the weaning phase (43). Interestingly, Peng et al. (20) studied the effect of the administration of 750 and 1500 mg/kg of Pot-ZnO on circulating immunoglobulin levels at day 14 in early weaned piglets. They found that 750 mg/kg and 1500 mg/kg of Pot-ZnO can enhance IgG levels comparable to a pharmacological administration of conventional ZnO (3000 mg/kg). Moreover, Liao et al. (44) showed that 320 mg/kg of cupreous N-carbamylglutamate chelate could trigger the immune response of nursery piglets, which was comparable to the supplementation of 650 mg/kg of CuSO_4_. Therefore, alternative formulations of both Zn and Cu appear to be as effective as conventional ZnO and CuSO_4_ in modulating immunoglobulin levels, but at lower dietary inclusion rates. On the other hand, this study demonstrated that the administration of 300 ppm of Pot-ZnO and 140 ppm of Cu_2_O showed a higher level of serum IgA compared to the PC group. Interestingly, the PC group was characterized by numerically lower values than the EU and Non-EU^+^ groups. Furthermore, no differences were observed among groups in IgG and IgM levels. Hence, these results are in line with previous findings and highlight that Pot-ZnO and Cu_2_O can efficiently modulate the systemic immune response of piglets when compared to pharmacological administration of ZnO throughout weaning.

DAO and L-lactate have been previously indicated as circulating biomarkers of intestinal permeability in weanling piglets (45). DAO is present within the apical fraction of intestinal villi, and its increase in the bloodstream can be related to the disruption of the intestinal barrier (46). Conversely, L-lactate is derived from anaerobe metabolism and could be linked to the development of specific microbial niches in the gut environment (47). Long et al. (18) showed that piglets fed 500 mg/kg of Pot-ZnO had a higher DAO level compared to a PC fed 3000 mg/kg of ZnO. In contrast, the administration of 200 and 500 mg/kg of Pot-ZnO did not decrease serum DAO levels when compared to pharmacological administration of ZnO (3000 mg/kg) at day 28 after weaning (19). In our study, L-lactate levels were similar among the treatment groups. Nonetheless, Non-EU^−^ group registered a higher DAO level compared to the PC and Non-EU ^+^ groups. In addition, EU and Non-EU^+^ treatment groups did not differ in DAO levels compared to the PC group. Perhaps, intestinal permeability may be influenced by a homeostatic regulation of Zn and Cu metabolism, which could be driven by the supplementation of a more balanced ratio of these trace elements when provided through Pot-ZnO and Cu_2_O.

In our study, fecal samples collected at day 14 revealed a significantly higher concentration of zinc in PC samples compared to the other groups. Zn intake above the required level can reduce the efficiency of Zn absorption, leading to increased fecal excretion (48). Wang et al. (49) highlighted a reduction in Zn fecal excretion by supplementing piglet diet with 400 mg/kg and 800 mg/kg of ZnO nanoparticles instead of 3000 mg/kg of conventional ZnO. Therefore, the presented results are in line with other studies comparing the ZnO pharmacological dosages and lower levels of alternative ZnO sources. In this study, the PC group demonstrated higher levels of fecal Cu than both EU and Non-EU^+^ at day 14. This result can be explained by the absence of Cu supplementation in PC during the first phase. In addition, Non-EU^+^ revealed a higher Cu concentration than Non-EU^−^ at day 14, which is in line with the Cu content supplied during the 1–14 d phase. Interestingly, the EU group showed higher Cu values than the Non-EU^−^ group at day 14 despite the same Cu supplementation. This difference can be explained by Cu’s endogenous regulation, which can influence Cu excretion in pigs (50). During the second phase of the trial, lower Zn/Cu ratios were administered. Adapting Zn and Cu supplementation can be useful for a balanced trace element metabolism and, consequently, a lower environmental output (51). In addition, when supplemented at an equal level (100 mg/kg), zinc concentration in the excreta does not differ between ZnO and Pot-ZnO formulations (52). Therefore, the results registered during the second phase of the trial were expected, as excretion is driven by trace element intake, and trace mineral retention in pigs is low (53). Once excreted, Zn and Cu can accumulate in agricultural soils following prolonged applications of swine manure, increasing environmental pollution (4, 54). In addition, Zn levels in liquid pig manure are strongly associated with diffusion of antimicrobial resistance (55). Thus, heavy metal compounds can bioaccumulate throughout the food production chain, contributing to the spread of antimicrobial resistance, which represents a potential risk for human health (56). Therefore, given the results discussed above, it is reasonable to consider that balancing Zn/Cu ratios supplemented through Pot-ZnO and Cu_2_O may represent an ideal strategy to align with sustainable farming practice and One-health principles.

ALP is a key indicator linked to pivotal biological pathways (57). It is a metalloenzyme with Zn as an integral component (58). Intestinal ALP may reflect the damage of the intestinal barrier as it has been linked to tight-junction modulation and, consequently, gut permeability (59). Martin et al. (60) revealed that jejunal ALP activity increases on administration of pharmacological levels of Zn (2500 mg/kg). In this study, the administration of different dietary Zn/Cu ratios through Pot-ZnO and Cu_2_O or a pharmacological dose of ZnO did not impact intestinal ALP levels. However, the literature remains inconsistent regarding the effects of Zn/Cu ratios on intestinal ALP activity in weanling piglets. Therefore, further evaluations are needed to elucidate the interaction of Zn/Cu ratios with intestinal ALP.

Intestinal sIgA is produced by plasma cells in the lamina propria. Once released, sIgA binds antigens, inhibiting the proliferation and pathogenic effects of potentially harmful bacteria, thereby contributing significantly to host defense (61). Previous studies have shown that alternative forms of zinc can enhance sIgA production in avian species and in the jejunal mucosa of postweaning piglets (62, 63). In this study, the PC group exhibited higher jejunal sIgA concentrations than Non-EU^−^ group, but not when compared to the EU and Non-EU ^+^ groups. These results suggest that a balanced supplementation of Zn/Cu through Pot-ZnO and Cu_2_O can positively influence local immunity throughout weaning.

In a previous study, Long et al. (64) detected changes in ileal digesta when administering 500 mg/kg of Pot-ZnO, which revealed a lower Chao1, observed, and Simpson indexes than a positive control group fed 3000 mg/kg of conventional ZnO. In contrast, they underlined a lack of difference between positive control and 500 mg/kg of Pot-ZnO in alpha diversity indexes evaluated in colonic digesta. In this study, Non-EU^−^ fecal samples collected at day 14 highlighted a reduction in observed, Shannon, and Simpson metrics compared to the EU group. Furthermore, beta diversity highlighted a significant separation in terms of microbial composition at day 14 among the treatment groups. Supplementing 200 and 500 mg/kg of Pot-ZnO linearly decreased *Escherichia coli* and *Clostridiaceae* counts in postweaning piglets’ digesta compared to 3000 mg/kg of conventional ZnO (19). The study demonstrated an abundance of *Clostridium sensu stricto* 1, 6, and 13 in the PC group compared to the other groups at day 14. *Clostridium sensu stricto* genera have been demonstrated to be useful biomarkers of intestinal health (65, 66). In addition, *Sutterella* abundance was lower in the EU, Non-EU^−^, and Non-EU^+^ fecal samples collected at day 14 compared to the PC samples. *Sutterella* represents a group of gram-negative bacteria previously linked to gastrointestinal disorders and IgA-degrading activity. It is negatively correlated to anti-inflammatory cytokines (66, 67). Our results aligned with previous findings and evidenced the capacity of Pot-ZnO and Cu_2_O to positively modulate gut microbiota by reducing the presence of potentially harmful genera. In addition, it was observed that Pot-ZnO and Cu_2_O, when administered within the European limitations (EU), increased the abundance of *Prevotella*, whereas the pharmacological ZnO administration (PC) favored different *Prevotellaceae* groups at day 14. Both *Prevotella* and *Prevotellaceae* have been linked to beneficial effects such as production of endogenous enzymes for carbohydrate digestion, better growth performance and diarrhea control, and development of mucosal immunity in pigs (66, 68). However, *Lachnospiraceae* abundance was higher in the EU group than in the PC group. *Lachnospiraceae* was linked to better carbohydrate digestion and energy and short-chain fatty acids production in the gut environment, with positive effects on gut health in pigs (66, 69). In addition, *Fusicatenibacter* was particularly prominent in the EU samples. This genus was previously associated with volatile fatty acids production and reduced intestinal inflammation (70). Therefore, the study illustrated that the balanced administration of Zn and Cu via Pot-ZnO and Cu_2_O within European inclusion levels was associated with a reduction of potentially harmful genera and increased abundance of gene sequences related to beneficial effects on the gut health of weanling piglets. However, future studies should focus on identifying the mechanisms behind the modes of action of the tested molecules on the intestinal microbiota. The results collected at day 28 indicated mild changes in microbiota composition among groups. This result can be linked to the microbial stability of the gut environment, which can be gradually achieved from day 10 after weaning (71). Hence, considering the differential abundances data along with the absence of effects in terms of alpha and beta diversity, it is reasonable to assume that the end of the trial was characterized by the presence of more stable enterotypes among groups.

## 5 Conclusion

Supplementing Zn and Cu through Pot-ZnO and Cu_2_O within European limitations can result in appreciable effects on gut health parameters and local immunity markers of weanling piglets. Indeed, during the first 2 weeks post-weaning, EU treatment enhanced the abundance of beneficial microbial niches associated with a healthy gut environment, as also evidenced by the results obtained on gut permeability and local immunity. This nutritional strategy may also contribute to reduced Zn and Cu excretion into the environment if routinely applied in commercial farms outside Europe, where higher trace element inclusion levels are still permitted. Nevertheless, future studies should investigate the optimal Zn/Cu ratios when using Pot-ZnO and Cu_2_O under practical farming conditions and across the entire weaning period, in order to further refine trace element supplementation strategies. In conclusion, the present study demonstrates that balancing Zn and Cu ratios via Pot-ZnO and Cu_2_O supplementation represents an effective approach to enhance gut health and immune function in weanling piglets.

## Data Availability

The original contributions presented in the study are publicly available. Sequencing data are available in the NCBI Sequence Read Archive (SRA) under the accession number PRJNA1378906.
